# Breaking Down the Compatibility Problem in Smart Homes: A Dynamically Updatable Gateway Platform

**DOI:** 10.3390/s20102783

**Published:** 2020-05-14

**Authors:** Linh-An Phan, Taehong Kim

**Affiliations:** School of Information and Communication Engineering, Chungbuk National University, Cheongju 28644, Korea; linhan@cbnu.ac.kr

**Keywords:** Internet of Things, smart home, gateway, device profile, compatibility, heterogeneous networks

## Abstract

Smart home is one of the most promising applications of the Internet of Things. Although there have been studies about this technology in recent years, the adoption rate of smart homes is still low. One of the largest barriers is technological fragmentation within the smart home ecosystem. Currently, there are many protocols used in a connected home, increasing the confusion of consumers when choosing a product for their house. One possible solution for this fragmentation is to make a gateway to handle the diverse protocols as a central hub in the home. However, this solution brings about another issue for manufacturers: compatibility. Because of the various smart devices on the market, supporting all possible devices in one gateway is also an enormous challenge. In this paper, we propose a software architecture for a gateway in a smart home system to solve the compatibility problem. By creating a mechanism to dynamically download and update a device profile from a server, the gateway can easily handle new devices. Moreover, the proposed gateway also supports unified control over heterogeneous networks. We implemented a prototype to prove the feasibility of the proposed gateway architecture and evaluated its performance from the viewpoint of message execution time over heterogeneous networks, as well as the latency for device profile downloads and updates, and the overhead needed for handling unknown commands.

## 1. Introduction

Smart homes is one of the most promising applications of the Internet of Things (IoT). However, the adoption rate of smart homes is still low despite substantial research about this technology in recent years. As a recent study [1] explains, there are several barriers to the mass adoption of smart home technology, and one of them is technological fragmentation within the smart home ecosystem. Many connectivity technologies (protocols) can be currently used in a connected home [2], which is confusing for smart home consumers. To solve this fragmentation problem, researchers and companies tend to advocate one of two approaches: (1) creating a universal protocol [3] that can be used for all devices and all use cases in smart homes and (2) developing a gateway supporting many protocols that acts as a central controller for all smart devices in the home.

Regarding the first solution, there has been a competition among wireless protocols in smart home networks: many new protocols have been suggested, tested, and even deployed commercially. However, ultimately, it is unlikely that there will be a clear winner in this battle; as different protocols are currently used in smart home networks [4]. Because each protocol has its own advantages and disadvantages, different protocols are best for different purposes and usages. Thus, it is difficult to design an all-in-one protocol for smart homes. Hence, the most suitable solution is to design a gateway to handle diverse protocols as a central hub in a home [5]. This solution seems preferable as more products appear on the market nowadays. However, this solution leads to another issue for manufacturers, which is the compatibility problem. Because of the various smart devices on the market, to support every possible device is a big challenge. This is why each gateway manufacturer always maintains a compatible device list for their gateway.

In Table 1, we survey some popular commercial smart home gateways on the market and the number of devices that they support. These numbers are impressive, but they still cannot cover all available smart devices on the market. In other words, there are many devices that still cannot work with these gateways. If a customer wants to use these devices in his/her house, he/she must use more than one gateway (hub). This makes control less convenient and limits consumers’ choices when buying smart devices. In the future, the compatibility problem will become more critical since increasing numbers of smart devices will appear daily. Until this problem is solved, consumers will have to face the difficulty of choosing a smart home system and smart devices, and as a result, the prevalence of smart homes will remain hard to achieve in the near future.

In this paper, we propose a software architecture for smart home gateways to tackle the aforementioned compatibility problem. Our gateway supports multiple protocols, unified control over heterogeneous networks, and allows developers to create applications to control diverse types of devices without any in-depth knowledge of the underlying protocols. In detail, we create a virtual device for each physical device in the home and provide standard RESTful APIs for interacting with this virtual device. In summary, this paper aims to propose a mechanism to easily support new devices that will appear in the future.

To satisfy this requirement, the gateway basically needs to know all the information and functions about a new device. This information comprises what is called a *device profile* in this paper. A device profile is a file that contains information about the device (e.g., manufacturer or model name), its functions, and how it can be controlled. If the gateway lacks a device profile, it cannot completely control a new device. We introduce a *Device Profile Handler* module to dynamically download and update the device profile every time a new device is connected to the gateway. This mechanism enables the gateway to be able to work with new devices; therefore, it can solve the compatibility problem. In addition, this approach does not require any modifications in either the protocol or the device; hence, it can be applied to current devices on the market.

The rest of this paper is organized as follows: in Section 2, we review related studies on smart home gateways. Section 3 explains what the compatibility problem is and why it happens in smart homes in detail. In Section 4, we describe how we designed the software architecture to achieve a dynamically updatable gateway supporting unified control over heterogeneous networks. We also present a prototype to prove our concept in Section 5 and evaluate its performance in Section 6. Finally, we discuss the feasibility of the proposed solution in Section 7 and conclude this paper in Section 8.

## 2. Related Work

### 2.1. Smart Home Gateway

Since smart home technology has emerged, numerous studies have been conducted on different aspects such as its architecture, communication protocols, application services, and security. Regarding the architecture studies, most early studies [12,13,14] as well as recent works [15,16] use OSGi-based architecture to develop smart home gateways that support heterogeneous networks. These studies have achieved the initial goal of a gateway—to make different protocols work together in the home network. For example, Kim et al. [12] proposed a comprehensive software architecture for supporting the seamless integration of devices and services and providing access control in smart homes. This study also presented a process to discover and install new devices using bar-codes and smartphones. In spite of that, it does not clearly address how to fully support a new device type. Overall, prior studies have been successful in making a platform for smart home gateways and created a fundamental research basis for further studies. However, they do not present any solution for handling the variety of devices on the market.

Another approach to making a smart home gateway is leveraging existing devices in a home such as a TV set-top box [17,18] or smartphone [19,20]. For instance, there have been studies [17,18] that proposed the integration of a smart home gateway and a TV set-top box to provide a unified experience from the smart home and TV services, and hardware implementations have been developed to prove the feasibility of this idea. These studies have opened a new approach to design a smart home gateway. Meanwhile, smartphone-based solutions [19,20] utilize the smartphone hardware with multiple radio interfaces (e.g., Wi-Fi and BLE) to communicate with different devices. Although smartphones are very popular today, they are not always located at a fixed place in the house and cannot be the main gateway of the smart home system.

In addition to the studies on architecture, there has been an increasing number of studies that implemented a smart home system based on specified protocols. The majority of these studies have targeted Wi-Fi [21,22,23], ZigBee [24,25,26], or combined ZigBee and other protocols such as Bluetooth [27] or Power-line communication [28]. In our previous work [29], we implemented a gateway using Z-Wave, which is one of the most popular protocols for smart home systems nowadays. Although Z-Wave provides diverse types of smart home devices, that study has a fundamental limitation because it supports only one protocol. Therefore, a multi-interface gateway is a mandatory requirement to support the heterogeneous networks in a modern smart home system.

In brief, Table 2 compares the previous and proposed gateways in terms of type of hardware design, heterogeneous network supported, unified control with API, and target problem, proving that in spite of intensive studies on the gateway, how to handle the increasing number of IoT devices and types had to be still considered. This paper aims to fill the literature gap by tackling the compatibility problem between smart devices and gateways.

To make it clear, we do not aim to redefine the entire software architecture of a gateway or propose a new approach to implement a gateway in this work. Instead of that, we introduce a design of gateway architecture to cope with the exponential growth of smart devices. In fact, some components of our proposed architecture are based on previous studies because they are the core components of any gateway platform (e.g., the driver and database). In addition, we add a new module called the *Device Profile Handler* to implement our ideas. Unlike the OSGi-based architecture, which is written only in Java, the proposed architecture is not restricted to any programming language.

### 2.2. Device Profile

Device discovery is also a challenge in the age of IoT. A number of studies have proposed approaches to address this issue in smart home applications. For example, Kim et al. [30] proposed a device management and auto-configuration platform using the Message Queuing Telemetry Transport (MQTT) protocol. Another study [31] proposed a configuration framework leveraging upon the Constrained Application Protocol (CoAP) for IoT devices. Recently, a study [32] used the Device Description Language (DDL) to describe the metadata of a device and how to access its services. The descriptive metadata is embedded in each device and exchanged through the MQTT or CoAP protocols. Unfortunately, the MQTT and CoAP protocols only work on top of the TCP/IP protocol, which is not suitable for many non-IP devices. Protocols such as ZigBee, Z-wave, and Bluetooth are not IP-compatible, and it is impossible to control these devices through MQTT or CoAP protocols. In addition, several protocols designed to support seamless discovery of devices and services in the network are the Devices Profile for Web Services (DPWS) or Universal Plug and Play (UPnP). However, similar to MQTT, DPWS and UPnP also only work with IP devices. Additionally, UPnP has a security risk and is not recommended for IoT devices which are more vulnerable to cyber-attacks [33].

A recent study [34] recognized the compatibility problem in current smart home gateway systems. However, the approach proposed in this research requires an intermediate server to run on top of the other gateway software, which means that a user still needs more than one gateway in the home. Of all the open-source gateway software, openHAB [35] is the most famous software for home automation. In this software, we found that to add a new device type on the market, users must write configuration files manually. This task requires programming skills as well as deep technical knowledge, which is difficult for most normal customers. In brief, we still do not see any convenient way to support a new type of device in current smart home systems. In the meanwhile, our proposed solution can work with non-IP protocols such as ZigBee, Z-Wave. It also does not require device manufacturers to make any change in their products, all changes are made at the gateway. This means the proposed idea can be applied to existing devices on the market.

In summary, previous academic works focus on making a platform that combines multiple protocols together to support heterogeneous networks. Meanwhile, companies try to create closed ecosystems and only support a limited number of device types. However, over time, increasing numbers of new smart home devices will be invented, which will lead to more complicated use-cases (applications) that can be defined to support new types of devices. That defines a new problem about compatibility which is usually ignored in previous studies. Clearly, the very first requirement of any gateway is to be able to connect to various devices. Hence, we need a solution to make it easy for a gateway to support new types of smart devices in the future, which would automatically increase the compatible device list for smart home systems. In this paper, we focus on presenting a solution to solve the compatibility issue completely. Therefore, the main contribution of this paper is to build a smart home gateway platform that can interact with any type of smart device, today or in the future.

## 3. Compatibility Problem

Most of the home gateway solutions on the market nowadays are a closed ecosystem, and the main problem of these solutions is device compatibility. In other words, when a customer decides to buy a new smart home device, they need to ask whether it will work with their gateway (because the new device may not). It is important to note that a modern smart home system may contain a number of different device types (e.g., lighting, switches, sensors, security devices, and other home appliances). Apparently, each device type has different features and purposes. Even devices in the same type can also have different functions. For example, one smart bulb could provide on-off and dimming functions, while another bulb might provide extra functions like changing color and blinking. Another example is sensor devices. There is a wide variety of sensors such as door, motion, temperature, and gas sensors. As a result, it will be increasingly difficult to support every new device in heterogeneous networks [36] owning to the proliferation of connected home devices.

To explain why the compatibility problem exists and why it is a huge problem of smart homes, we need to look at some characteristics of smart home devices. A smart home device is usually a resource-constrained device that has limited CPU, memory, and power resources. Because of these characteristics, smart home devices need to use low-power communication protocols such as ZigBee, Z-Wave, Bluetooth Low Energy (BLE), or Thread. Except for Thread, these protocols use a specific application layer in their design. In this case, all functionalities of a device (e.g., turn on, turn off, and send notifications) are specifically defined in the protocol and the developers have to follow the protocol specification to create an application.

The protocol’s inventors typically organize a set of related functions as an application profile. There are many application profiles defined for different types of devices. An application profile contains specific details about what information a device can communicate. Of course, besides the standard profiles, all these protocols also support option fields for manufacturers to define their own custom profiles for a special purpose or particular application. This protocol design strategy benefits the device maker because it makes it easier to develop applications. On the contrary, it imposes a processing burden on the gateway. In detail, a gateway needs to know all the application profiles that were implemented on every smart device (product), so that it can communicate with a variety of devices. Moreover, to provide a seamless experience for users through the control application, the gateway needs to know extra information related to device such as the product’s name, description, and image of product so that this information can be accurately displayed in the user interface of the control application.

This large amount of information is very important, but it cannot be completely embedded inside a device as well as exchanged through the communication protocols because of bandwidth limitations. For this reason, gateway manufacturers need to pre-implement the application profiles and information of all devices that they want to support on the market. In other words, the gateway must already know all the information about a device it will work with in order for it to be compatible with the device. As a result, there are two cases in this situation: (1) the gateway has already implemented all the application profiles of a new connected device and also knows what device it is controlling or (2) the gateway does not have enough information about a new connected device, so it cannot fully control this device. The second case is referred to as the compatibility problem that we want to address. If the gateway manufacturer wants to support new devices in the future, they need to update the software for the gateway. However, this process usually takes a long time and it depends on the development plan of each manufacturer.

Overall, the compatibility problem is real and is becoming more critical nowadays. Therefore, it needs to be resolved in the development of every gateway product. We believe that making a universal protocol will be hard to achieve in near future while the current closed ecosystems still have many limitations. Solving the compatibility problem would help accelerate the adoption rate of smart homes [37], and this is the main goal of this paper.

## 4. System Architecture Design

**Figure 1:**
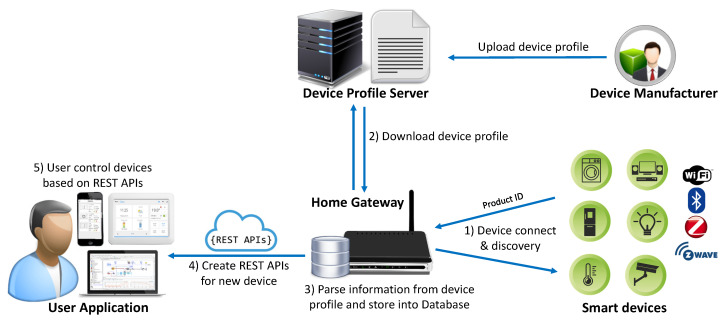
Components and operation of a smart home system.

A smart home system consists of various smart devices, a central home gateway, and a client application that enables the user to control all smart devices as shown in Figure 1. A home gateway is a hardware device with multiple integrated wireless interfaces; thus, it is able to communicate with a variety of devices using different protocols. In this section, we describe the software architecture that enables a unified control over heterogeneous networks and supports dynamic updates of device profiles for new devices.

### 4.1. High-Level Architecture

We illustrate the components and operation of our gateway system in Figure 1. First, every smart device in a home needs to be discovered and connected to a network that is formed by the gateway device (this step is also called pairing, joining, or bootstrapping). The procedure of discovery and connection is different according to each protocol, and it may require some actions from the user (e.g., to push buttons or input code). Through this process, the gateway and device exchange some basic information such as manufacturer, device type (product) identification, and a list of the names of application profiles that are used in the device. The gateway can download the device profile according to the device type identification information. Note that the device type identification information can be different according to the underlying network protocols. For example, both ZigBee and Z-Wave use a unique 16-bit hexadecimal number to represent a manufacturer as well as device type, but this number is different in each respective protocol. Similarly, each protocol also provides a different mechanism to obtain the device type identification information.

**Figure 2:**
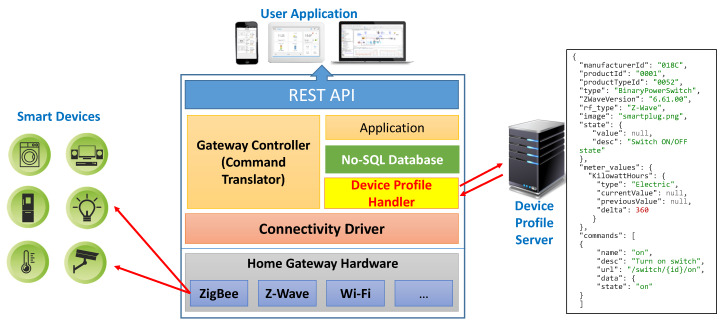
Software architecture of the smart home gateway.

In fact, device type identification information is mandatory to distinguish a specific device type (product) from others. If a new type of device is discovered, the gateway connects to the device profile server and downloads the device profile according to each device type (product). By parsing this information from the downloaded file and storing it in a database, the gateway is able to know all the details of application profiles and has a description of the new device. Thus, without any software update, the gateway still can provide correct RESTful APIs for client application to communicate with the new device. In addition, if the device makers want to add new features to their device, they can also update the corresponding device profile on the server. In this case, the gateway will download a new profile as it did the first time and dynamically update the RESTful APIs for the new features. The software architecture of the gateway is composed of multiple layers (modules) as shown in Figure 2. The detailed roles of each module are described in the next subsections.

### 4.2. Connectivity Driver

The *Connectivity Driver* is the lowest layer of the system. It provides a software interface to ensure that the gateway software can interact with the hardware. Gateway hardware usually consists of a single-board computer, which includes a micro-processor, storage, and communication modules (e.g., Wi-Fi, ZigBee, Z-Wave, or BLE). Each type of communication module needs a particular driver to connect the hardware and the upper software layer. The driver is responsible for communicating with physical devices.

### 4.3. Device Profile Handler

The *Device Profile Handler* is the key module of the proposed architecture that helps to solve the compatibility problem. It is in charge of downloading and updating the device profile from the server. When a new device type is connected to the gateway, the *Device Profile Handler* immediately connects to the *Device Profile Server* and downloads the corresponding device profile of the new device. Note that we assume that the profile servers are owned and maintained by the manufacturers in a distributed manner and the location of the profile server is known by the gateway. The device profile server architecture and the resolution system required to determine the location of the profile server are out of scope of this paper. However, it is possible to apply various existing systems. For example, GS1 [38] provides a distributed profile server architecture and ONS-based resolution system.

After downloading a device profile, the *Device Profile Handler* will read the information from the downloaded file and store it into a database. This process is only performed once for each type of device, and the profile file is permanently stored in the gateway’s storage. Thus, if the gateway connects with the same device type in future, the *Device Profile Handler* only needs to read its information from a local file. This procedure can increase the performance of the system. If a manufacturer updates a device profile on the server, the *Device Profile Handler* will download a new version of the device profile and automatically add the changes into the system.

The second role of the *Device Profile Handler* is to act as a bridge between the *Driver* and *Controller* module to deal with a new type of device on the market. The compatibility problem comes from the fact that the gateway cannot know how to handle unknown application profiles or custom profiles. In detail, the gateway needs to know what this device is and how to use all the functions implemented in the new device. More technically, each application profile contains a set of functions for the smart device, and each function is represented by value (in bytes) exchanged between the gateway and device. In fact, the gateway and device exchange a stream of bytes that forms a command. From the command, we can obtain meaningful information (e.g., “turn on a light” or “report the current temperature”) by interpreting the command according to a specific format. Without the information from the application profile, the gateway cannot know how to interpret a command.

**Figure 3:**
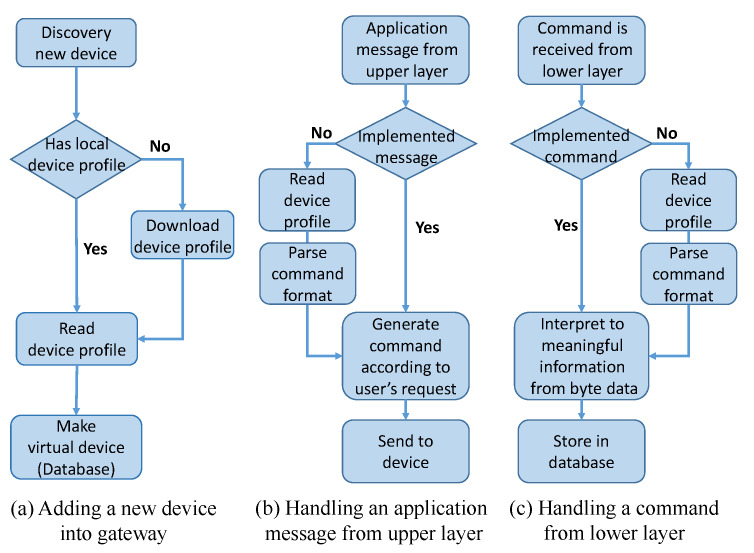
Process flow of the gateway for three cases: (**a**) adding new device to the gateway, (**b**) handling a command from an upper layer, and (**c**) handling a command from a lower layer.

We solve this problem by storing the specifications of all application profiles in the device profile and then using them in the gateway’s operation. Figure 3 illustrates the process flow of a gateway and the *Device Profile Handler* in three cases: (a) adding a new device to the gateway, (b) handling an application message from the upper layer, and (c) handling a command from the lower layer. As mentioned in Section 4.1, for case (a), the gateway downloads the corresponding device profile if a new type of device is discovered, as shown in Figure 3a. In cases (b) and (c), we assume that the gateway is communicating with a new device type and it does not implement commands for this new device. In these cases, the *Device Profile Handler* reads the device profile from the database and handles the unknown commands. For instance, Figure 3b shows the procedure for handling an application message from the upper layer (a user’s request). To send the user’s request to the device, the gateway needs to generate a correct command according to the user’s request and command format. If this command is not implemented, the gateway can parse the command format by reading the device profile. Here, we assume that the request from the user is authorized to perform on the device. Advanced functionalities of a typical IoT gateway such as user authentication, access control, and data validation can be implemented in the *Controller* or *RESTful API* layers. The implementation of these functions is discussed in Section 4.4. It is worthwhile to note that the *Device Profile Handler* is the core function of this study and the rest modules can be implemented the same as a traditional gateway providing fruitful functions based on stateful information.

In contrast, in Figure 3c, if a command is received from a lower layer (the device), the gateway needs to interpret meaningful information from the byte data. To do that, the command format is necessary, and it can be parsed from the device profile, similar to the process shown in Figure 3b. To clarify, a device profile is like a guidebook that helps the gateway to communicate with a new smart device. With this mechanism, although the gateway does not fully implement all the application profiles (commands) of the new device, it still able to control new kinds of devices without a software update.

**Figure 4:**
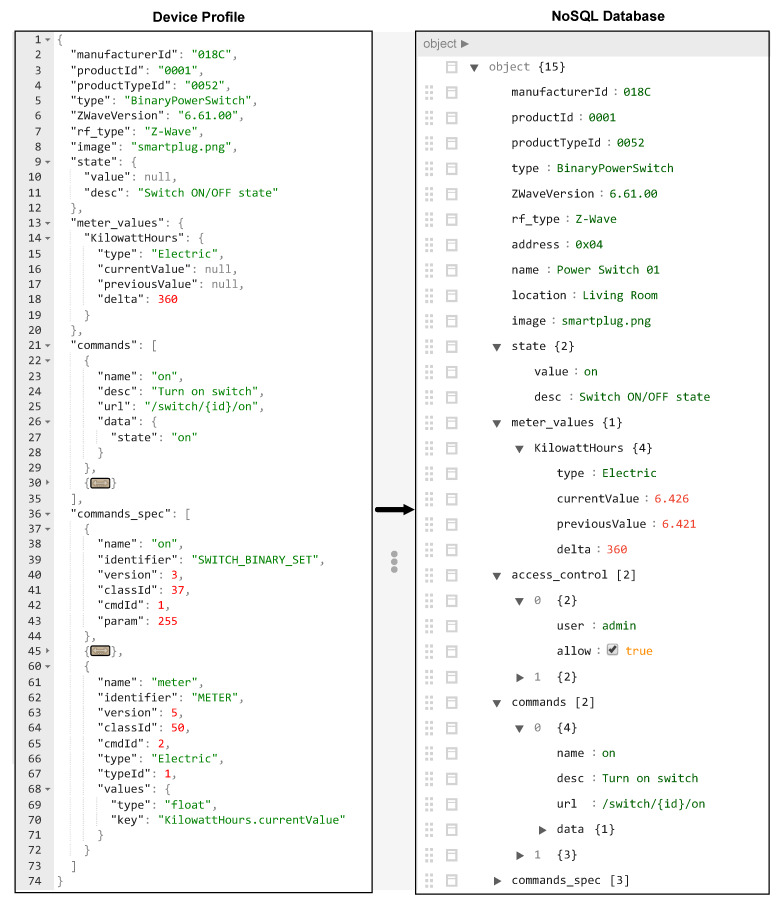
All information of a device is stored in the NoSQL database as a virtual device.

In our architecture, we use JSON as the data format for the device profile. The JSON format is sufficient for describing device information and has been used in other studies [14,34]. Because other modules of the gateway software (i.e., NoSQL database and RESTful API) also use JSON format, this design reduces the cost for converting data between components. Another advantage of JSON is that it is a lightweight and very readable format. An example of a device profile of a Z-Wave switch is shown in Figure 4. Basically, the device profile is composed of two parts: general and specific information. The general information includes the identification and description of the device (lines 2–8 in Figure 4). Based on that information (i.e., device type, protocol type), the gateway can determine how to properly handle the specific information in the device profile. For example, the specific information in the device profile of a Z-Wave device can be interpreted differently compared to that in a ZigBee device.

Meanwhile, the specific information includes status and commands (functions) that are used to communicate with the device (lines 9–74 in Figure 4). The specific information can be categorized into three parts such as state information (lines 9–20 in Figure 4), commands (lines 21–35 in Figure 4), and the specification of status and commands (lines 36–74 in Figure 4). The first part describes the template to store state and all sensing information that are frequently collected from the device. This signifies that all information about the device can be obtained by control applications through RESTful APIs. The second part describes all available commands of the devices and the corresponding RESTful APIs to use these commands. This helps the users to fully control their devices in the smart home system. Note that the semantics of each status information and commands (i.e., Switch ON/OFF state, Turn on switch) can be provided through the description field. Moreover, additional information can be defined after the device is connected to the gateway, such as device name (i.e., power switch 01) and device location (i.e., living room), as shown in Figure 4. Therefore, semantic models can be built based on the information in the device profile and NoSQL database. Finally, the third part describes in detail how to construct each command of each device. More precisely, this part provides the guidance to build a command, to interpret the data that receives from a device, and to store the result in the template that is defined in the first part.

As explained previously, a command is formed by a set of data as defined in the protocol. Therefore, the gateway only needs to combine data in the device profile to construct a defined command. In an opposite way, the data that receives from a device must be interpreted to a human-readable data (i.e., string, number) and stored in the database. The device profile contains information such as the data type, mapping values, and the result destination so that the gateway can precisely interpreted the information from the device. For example, Figure 4 shows that the gateway must convert the data periodically reported from a smart switch to a float number to present the consumed energy of that switch. Note that the number of attributes and the values of them in the device profile may vary depending on the type of the device or the connectivity protocol. However, it is reasonable to assume that the structure of the device profile is known by all manufacturers in advance. Moreover, the JSON format has sufficient flexibility to support various types of device products and the connectivity protocols. Thus, the JSON-based device profile is expected to be widely used in future applications and scenarios.

### 4.4. NoSQL Database

A NoSQL database is a new kind of database system that can replace traditional relational databases. Information such as the description, status, and features of each device are very different, so it is complicated to store in a traditional database. However, a NoSQL database can store different varieties of data, particularly unstructured data. Because of these characteristics, a NoSQL database was used in the gateway’s system. Moreover, to provide unified control over heterogeneous networks, we need to abstract each physical device as a logical device (called a *virtual device* in our platform). Virtual devices are stored in the NoSQL database, as shown in Figure 4, and the user application can only interact with these virtual devices via standard RESTful APIs.

Using the virtual device allows developers to manipulate any device type without having a deep understanding of the underlying protocol or hardware design of the device. Also, it is easy to create additional services or applications based on the virtual device approach. For example, a security feature such as access control [39] can be built by adding user permissions information into virtual devices. Meanwhile, an intrusion detection service can be developed by combining time-series data of virtual devices and machine learning techniques [40]. Note that preventing security threats that occur at lower layers in the smart home system (e.g., hardware, network) typically depend on the design of underlying protocols and not that of the gateway [41]. Most smart home protocols provide built-in security features, such as node authentication, message encryption, and key management, independently from the gateway. In brief, connectivity protocols provide security features for communication between devices and the gateway, while secure communication between the gateway and users will be provided by high-level security services that are installed in the gateway. These security aspects are out of scope of this paper and can be handled by other studies.

Another important application that can be leveraged on the virtual device approach is device-to-device communication. Since the information of physical devices can be proactively or reactively updated to the gateway, virtual devices exactly reflect the current status of physical devices. Therefore, interaction among heterogeneous devices in the system can be performed partially at the gateway level through virtual devices. This allows to reduce the communication overhead between smart home devices as well as increase the scalability of the gateway. For example, an actuator can access sensing data of a sensor by simply querying the database without directly communicating with that sensor. Apparently, several complicated modules, such as protocol translator and message broker, are needed to achieve a seamless interoperability in the IoT system [42]. In addition, stateful applications, such as asynchronous event notification, publish/subscribe messaging, and cloud-based data analysis/storage services, can be developed by utilizing the information of virtual devices in the NoSQL database. For example, by integrating an open-source publish/subscribe framework [43], the gateway can act as a broker between message producers and message consumers. In other words, diverse sensor data from the devices can be stored and analyzed at a cloud server, while the external users or devices can remotely query the data through cloud services. This indicates that the gateway needs to be equipped with an additional publish/subscribe framework in addition to the proposed gateway architecture. However, the detailed design and implementation of these applications depend on the gateway vendors, and these processes are independent of the core gateway architecture described herein.

### 4.5. Controller

The role of the *Controller* is to translate commands from a user application into the respective command in the driver for each protocol. From an application viewpoint, developers do not need to know the details of underlying protocols because they only interact with virtual devices via RESTful APIs. All requests from the RESTful API are passed to the Controller, which detects which device the user wants to control, which protocol this device uses, and how to translate the user’s command to the corresponding protocol’s command. For example, if a user wants to turn off a kitchen light, they press the off button in an application. The application then sends an HTTP request to the gateway and information from the RESTful API passes to the Controller. The Controller detects the network address of this kitchen light from the database and knows that this light uses the ZigBee protocol. Finally, the Controller requests to send the light-off command from the ZigBee Driver to the physical kitchen light. After that, the driver takes care of the communication between the gateway and device.

### 4.6. RESTful Web Service

The RESTful web service is a set of standard APIs that are used for communication between control applications (client) and a gateway (server). A client application can be a traditional desktop, mobile, or web application. Since client applications are run on powerful devices, it is suitable to implement RESTful APIs over HTTP protocol. With the standard APIs, developers can customize the control application for a particular customer. To provide unified control over heterogeneous networks without any in-depth knowledge about real devices, we define all APIs based on the types and functions of devices. Table 3 shows examples of RESTful formats that are used in the gateway. With each type of device, we predefine common APIs, for example, a light device definitely has an on-off command in its APIs. Moreover, based on a device profile, the gateway can provide custom APIs for each device. For example, a special bulb (light) might have an API to provide the current temperature in the home.

The RESTful web service has become more popular because of its advantages, such as simplicity, better performance, and variety of data formats compared to the SOAP-based web service [44]. Since the RESTful web service also supports JSON format, it is convenient to exchange data among modules in the gateway. Furthermore, separating the control application and the gateway allows each component to be developed independently, which can reduce the development time of the gateway product. In case the manufacturers open the REST APIs publicly, it is easy for a third-party to develop a control application based on RESTful APIs thank to its simplicity. In summary, using the RESTful web service is beneficial to the development of a smart home gateway.

## 5. Prototype Implementation

**Figure 5:**
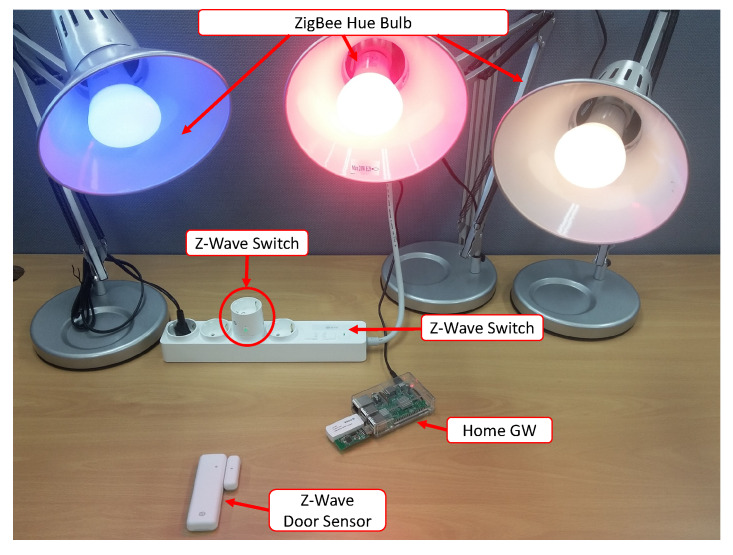
Smart home testbed with Gateway, ZigBee, and Z-Wave smart devices.

A prototype was implemented to prove the feasibility of our smart home architecture. Our testbed consists of a home gateway, six real smart devices, a device profile server, and a desktop application that acts as a user application, as shown in Figure 5. In this testbed, we evaluated all the gateway related functions from device discovery and profile downloading from the server to updating the device profile to the database (virtual device) in order to create RESTful APIs for a client application.

To build the gateway hardware, we used a single-board computer that has the Linux OS already installed. To support the ZigBee, Z-Wave network, we used two USB dongles acting as the ZigBee coordinator and Z-Wave controller, respectively. The single board also supports Wi-Fi and BLE, so our gateway hardware can support four protocols. Note that the goal of the proposed architecture is to enable the gateway to dynamically update the device profile for a new type of device. However, it does not mean that the gateway can be updated for a new type of network protocol since adding an underlying protocol into the gateway requires a hardware interface as well as relevant software modules, such as connectivity driver and controller. Therefore, we assume that the gateway is already equipped with the latest version of networking protocols that a new device works on.

**Figure 6:**
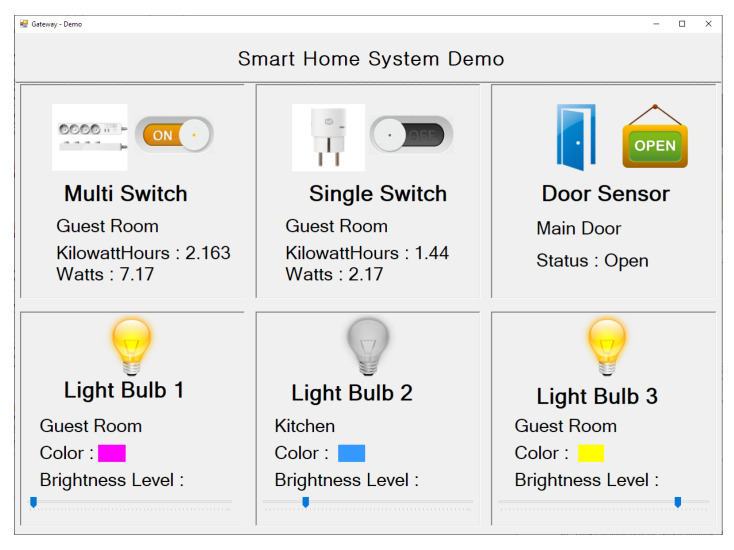
Smart home client (user) application on a PC.

Regarding the software implementation, the gateway software was written in Java. To implement the driver for Z-Wave and ZigBee (Connectivity Driver), we used two Java open-source software libraries, WZWave [45] and ZigBee for Java [46], respectively. A MongoDB database system [47] was chosen to implement the NoSQL database. To create a RESTful web service in Java, we used the Jersey framework [48]. In addition, we used PHP to implement a simple device profile server. Finally, to control the smart devices through RESTful APIs, we created a desktop application, as shown in Figure 6. There are no restrictions on making user applications, so developers can create any kind of web, mobile, or desktop application, and all of them can easily work with the RESTful APIs.

## 6. Evaluation

We evaluated the performance of the prototype with respect to message execution time over heterogeneous networks, the latency for downloading and updating, and the handling of an unknown command from the gateway to a device and vice versa.

### 6.1. Application Message Execution Time

**Figure 7:**
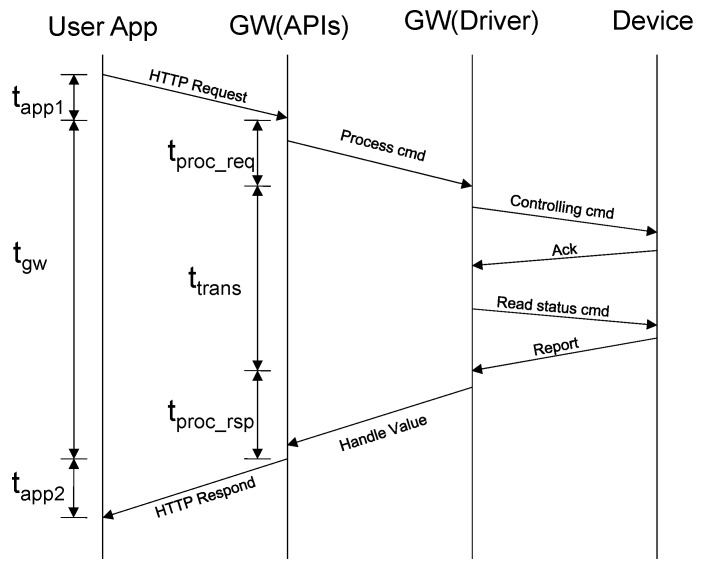
The runtime for executing one command from the user application.

Application message execution time is the most important criterion for evaluating the operating performance of a gateway system. The execution time for a user’s operation is calculated from the point at which a user executes a command on an application until they see the result for this command through the application display. For instance, a user wants to turn off a light, he/she pushes the OFF button of the application user interface. After that, the light is switched off and then the application displays the status of the light (OFF) according to the actual status of the device. The runtime of this procedure is divided into three phases, as shown in Figure 7: (1) *T${}_{app1}$* is the time required to transfer the request from the application to the gateway via a RESTful API, (2) *T${}_{gw}$* is the time needed for the gateway to handle the command from the user, and (3) *T${}_{app2}$* is the time needed for the application to receive the change from the gateway via a RESTful API and update its user interface. The times of *T${}_{app1}$* and *T${}_{app2}$* depend on the network condition and the design of the application. Thus, we measure them by focusing only on time *T${}_{gw}$*, which is from when the gateway receives the HTTP request until new values from the device are updated to the database.

As shown in Figure 7, we can divide the application message execution time further into (1) *T${}_{proc\_req}$*, which is the time for handling the HTTP request, (2) *T${}_{trans}$*, which is the time for transmitting a packet over the air, and (3) *T${}_{proc\_rsp}$*, which is the time for handling the response command and updating the new values in the database. The results of some different commands are listed in Table 4. There are some differences in the result of *T${}_{proc\_req}$* because we used different open-source software for implementing the Z-Wave and ZigBee drivers. However, in general, the total time the gateway needed to process everything is only about 250–300 ms. This amount time is fast enough that a user does not perceive any delay in the system.

### 6.2. Downloading and Updating Device Profiles

**Figure 8:**
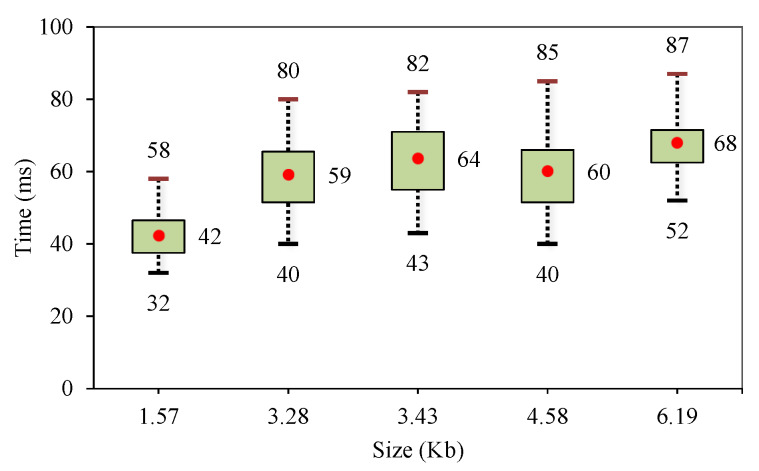
Total time for downloading and updating device profile.

The gateway needs to download the device profile whenever it connects with a new type of device (new product). When a smart home is composed of many devices that have the same product type, the gateway only needs to download the corresponding profile once. In our testbed, we used a PC to act as the device profile server and measured the time for downloading and updating the device profile in the gateway for different sizes of profile files. Both the PC and gateway were connected to the local network. The test case was repeated 15 times and the result is shown in Figure 8, which shows the average, maximum, and minimum values of each profile’s size respectively. Generally speaking, the gateway only needs under 100 ms to download and update a real profile file (which is usually smaller than 10 Kb). In practice, the connection between a gateway and server is an Internet connection, and it does not take a long time to download a small file over the Internet.

It is worth noting that even a new type of device can successfully join a network assuming that it has proper information and permission for authentication and association of the underlying networking protocol. This proves that the gateway can communicate with a new type of device freely from a viewpoint of networking, but it cannot interpret the application messages due to a lack of device profile. Since our goal is to enable dynamic update of device profile, we focused on the total time for downloading and updating device profile as shown in Figure 8. In other words, we excluded the time consumed for the networking perspective from this measurement, since the discovery and connection to the network is a networking perspective as discussed in Section 4.1 and they vary depending on the underlying network protocol. In summary, the time measured in Figure 8 can be considered that taken for learning the device profile on the application level.

### 6.3. Handling Unknown Commands with the Device Profile

**Figure 9:**
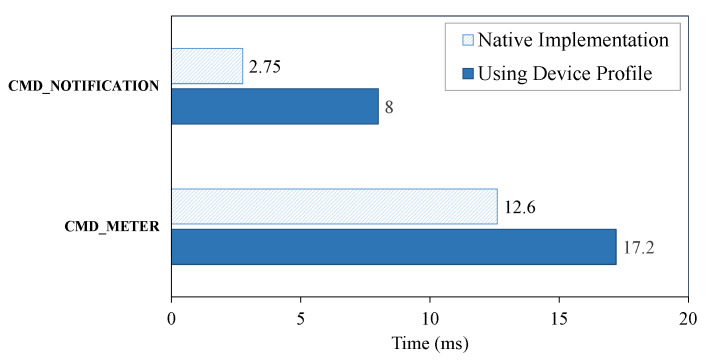
Comparison of the time needed to handle Z-Wave commands in two cases: native implementation and using a device profile.

We evaluated a special case of our gateway system—handling unknown commands with the device profile. Obviously, the gateway needs time to read a device profile and convert it to the relevant commands. In Figure 9, we compare the time needed to handle two commands (“notification” and “meter”) of the Z-Wave protocol for two cases: (1) a native implementation and (2) using a device profile. The notification command is used to notify the gateway of events that are reported from devices. The meter command is used to read the accumulated values in physical units from metering devices (e.g., water, gas, and electric meters). Both commands are very complicated because they cover many use cases and can be used by many device types.

To display the exact information about the device to the user, the gateway needs to understand the data received from a device, for instance like the kind of event or measurement the data represents or the value of meter. Note that the structure and meaning of the data are described in the specification document of the protocol. In case 1, we need to implement the usage of each command case-by-case in source code. In contrast, in case 2, we convert the usage of each command to the device profile, then guide the gateway to parse the information from the device profile. In both cases, the accuracy of the command is the same; however, the time needed for processing is slightly different. Although the result is still very positive, using a device profile takes about 5 ms longer than the native implementation. This amount time is short enough that a user is not able to recognize any difference in the system. Therefore, we conclude that the proposed gateway architecture provides a performance that is comparable to the native implementation while solving the compatibility problem effectively.

## 7. Discussion

The proposed gateway architecture breaks down the compatibility problem and allows consumers to freely choose smart devices from any manufacturer without technical knowledge about them. However, to realize the proposed solution, we need to resolve the assumption that device manufacturers provide the device profiles to a profile server so that the gateway downloads, parses, and interprets them dynamically. This section discusses the feasibility of this assumption as well as the advantages of the proposed solution.

Basically, the collaboration between device manufacturers and gateway manufacturers is reasonably necessary to achieve the compatibility regardless of the design of the gateway [49]. However, the proposed architecture can facilitate that collaboration and can be beneficial to both types of manufacturers. From the viewpoint of device manufacturers, providing device profiles allows their devices to be supported by multiple gateways simultaneously on the market. As a result, it helps to increase the accessibility and usability of devices produced by the manufacturer. Also, the device profile approach can be applied to launched devices on the market without requiring a firmware update. It is important to note that providing device profiles does not mean that all profiles are open, or that it provides less privacy and protection. It is possible to apply a secure mechanism so that only allowed individuals or vendors can search for and download device profiles from the profile servers. Therefore, we expect previously mentioned benefits will increase the number of IoT device manufacturers sharing their device profiles on profile servers.

The proposed architecture is fascinating to commercial gateway manufacturers, since it solves the compatibility problem in the proliferation of smart home devices in a totally open manner. Clearly, it is not a trivial task for gateway manufacturers to support the increasing IoT devices by themselves. Instead of manually implementing protocols for new devices within the gateway software, the proposed gateway solution can support new devices in a dynamic manner by downloading and interpreting device profiles from profile servers. Interestingly, these processes are totally transparent to the consumers. Therefore, we can conclude that the proposed solution significantly reduces gateway development and update time and provides compatibility for new IoT devices through the collaboration with device manufacturers.

In addition, there have been several standardization attempts [50,51] to solve the interoperability problem of IoT devices. In particular, W3C defines how IoT devices are described following the Thing Description specification [50] so that they can easily interact with each other. Similarly, a standardized description developed by an organization can facilitate the widespread use of the device profile across gateway platforms. Apparently, several aspects must be considered to achieve standardization. First, the standard development organization (SDO) must define a uniform set of attribute names (vocabulary) for the device profile, following the structure we discussed in Section 4.3. It is also necessary to specify whether each attribute is a mandatory or optional field. For example, the device type should be a mandatory field to identify the type of each device. Regarding protocol specific information, the SDO can provide a mapping guideline to clarify how to declare that information in the device profiles. For example, a “turn on” command in the ZigBee protocol may have different parameters compared with that of the Z-Wave protocol. Therefore, additional fields are needed to precisely describe and link each parameter from protocols into the device profile. Second, the data format of the device profile also needs to be clearly specified. The device profile can be represented by the JSON format like our proposed architecture. However, any other formats such as XML and YAML are also allowed if they can describe the variable set of attributes of the device. Finally, the standardization of the device profile description is expected to accelerate the adoption of the proposed gateway architecture to accommodate the proliferation of IoT devices.

## 8. Conclusions

The ultimate vision of a truly smart home system not only includes the automation and remote control of smart devices but also a combination of other smart applications such as healthcare [52], security [53], artificial intelligence [54], and ambient assisted living [55]. However, all these applications need seamless connections among all the smart devices through a central hub in the house to work more efficiently. In this paper, we proposed a software architecture for a gateway in a smart home system to support unified control over heterogeneous networks. In addition, based on device profiles, we can design a flexible and extensible gateway which can solve the compatibility problem in current smart home systems. We demonstrated the feasibility of our gateway platform and evaluated its performance with respect to application message execution time, device profile downloads and updates, and handling unknown commands with a device profile.

The proposed architecture can also serve as fundamental research for further studies because all devices are seamlessly connected to one central hub and mapped to a database as virtual devices. We believe this work can be applied in various gateway products and will leverage the development and adoption of smart home technology. For future works, we will consider to extend the evaluation testbed as well as develop advanced applications and services that utilize the device profile based smart home gateway. 

## Figures and Tables

**Table 1:** Commercial Gateway Products.

Vendor	Supported Protocols	Supported Devices
Samsung SmartThings [6]	ZigBee, Z-Wave, Wi-Fi	300+
Apple Homekit [7]	Bluetooth LE (BLE), Wi-Fi	130+
Wink Hub [8]	ZigBee, Z-Wave, BLE, Wi-Fi, Kidde, Lutron Clear	120+
VeraSecure [9]	ZigBee, Z-Wave, BLE, Wi-Fi, VeraLink	210+
Homey [10]	ZigBee, Z-Wave, 433 MHz, Wi-Fi, Bluetooth	70
HomeSeer [11]	Z-Wave, Wi-Fi, Serial, Ethernet	160+

**Table 2:** Comparison of previous studies about smart home gateway.

Gateway	Hardware Design	Hetero-Geneous Network	Unified Control with API	Targeted Problem
Kim et al. [12]	Independent gateway	Yes	Yes	Integration of heterogeneous, semantic interoperability
Sleman et al. [15]	Independent gateway	Yes	No	Hardware design of multi-interface gateway
Aloi et al. [19]	Smartphone based	Yes (Limited)	No	Mobile gateway solution
Moazzami et al. [20]	Smartphone based	No	No	Smartphone based home automation systems (focus on RESTful and SOAP-based smart devices)
Phan et al. [29]	Independent gateway	No	Yes	Z-Wave smart home gateway
Nugur et al. [21]	Independent gateway	Yes	Yes	IoT gateway for a cloud-based energy management system
Gavrila et al. [17]	TV Setup box based	Yes	No	An integration of smart home system to a TV set-top box
Proposed	Independent gateway	Yes	Yes	To tackle the compatibility problem between smart home devices and the gateway and support unified control over heterogeneous network

**Table 3:** Example Format of RESTful APIs in The Gateway.

API Path	Example	Method	Description
/api/device	/api/device	GET	Get all devices in system
/api/device/{:id}	/api/device/1	GET	Get information of device with id = 1
/api/device/light/:id/{:command}	/api/device/light/1/on	POST	Turn on device (light) with id = 1
/api/device/light/:id/{:command}	/api/device/light/1/color	POST	Set color for device (light) with id = 1
/api/device/security/:id/{:command}	/api/device/lock/3/unlock	POST	Unlock device (lock) with id = 3

**Table 4:** Time (Millisecond) for Executing a Command in Gateway.

Command	T${}_{\mathrm{proc}\_\mathrm{req}}$	T${}_{\mathrm{trans}}$	T${}_{\mathrm{proc}\_\mathrm{rsp}}$	Total (T${}_{\mathrm{gw}}$)
Z-Wave ON	80	151	10	251
Z-Wave OFF	80	151	10	250
ZigBee ON	12	209	11	232
ZigBee SET COLOR	14	233	12	259
